# Identifying quantitative operation principles in metabolic pathways: a systematic method for searching feasible enzyme activity patterns leading to cellular adaptive responses

**DOI:** 10.1186/1471-2105-10-386

**Published:** 2009-11-24

**Authors:** Gonzalo Guillén-Gosálbez, Albert Sorribas

**Affiliations:** 1 Departament d'Enginyeria Química, Universitat Rovira i Virgili, Av.Països Catalans 26, 43007-Tarragona, Spain; 2 Departament de Ciències Mèdiques Bàsiques, Institut de Recerca Biomèdica de Lleida (IRBLLEIDA), Universitat de Lleida, Montserrat Roig 2, 25008-Lleida, Spain

## Abstract

**Background:**

Optimization methods allow designing changes in a system so that specific goals are attained. These techniques are fundamental for metabolic engineering. However, they are not directly applicable for investigating the evolution of metabolic adaptation to environmental changes. Although biological systems have evolved by natural selection and result in well-adapted systems, we can hardly expect that actual metabolic processes are at the theoretical optimum that could result from an optimization analysis. More likely, natural systems are to be found in a feasible region compatible with global physiological requirements.

**Results:**

We first present a new method for globally optimizing nonlinear models of metabolic pathways that are based on the Generalized Mass Action (GMA) representation. The optimization task is posed as a nonconvex nonlinear programming (NLP) problem that is solved by an outer-approximation algorithm. This method relies on solving iteratively reduced NLP slave subproblems and mixed-integer linear programming (MILP) master problems that provide valid upper and lower bounds, respectively, on the global solution to the original NLP. The capabilities of this method are illustrated through its application to the anaerobic fermentation pathway in *Saccharomyces cerevisiae*. We next introduce a method to identify the feasibility parametric regions that allow a system to meet a set of physiological constraints that can be represented in mathematical terms through algebraic equations. This technique is based on applying the outer-approximation based algorithm iteratively over a reduced search space in order to identify regions that contain feasible solutions to the problem and discard others in which no feasible solution exists. As an example, we characterize the feasible enzyme activity changes that are compatible with an appropriate adaptive response of yeast *Saccharomyces cerevisiae *to heat shock

**Conclusion:**

Our results show the utility of the suggested approach for investigating the evolution of adaptive responses to environmental changes. The proposed method can be used in other important applications such as the evaluation of parameter changes that are compatible with health and disease states.

## Background

The emergence of design in biological systems was an unsolved problem until natural selection was established as the driving force for the evolution of such systems [1-4]. At the molecular level, the identification of design principles through controlled mathematical comparisons that evaluate different functional criteria in metabolic networks has led to a better understanding of adaptation and design emergence [5-12]. Such principles enable building new gene and metabolic networks that accomplish specific requirements which is the main goal of Synthetic Biology [13-15]. The identification of quantitative evolutionary constraints plays an important role in understanding the actual characteristics of biological systems [16,17]. In that sense, one may argue that the adaptive response of the cellular metabolism to different situations is ultimately shaped by physiological requirements that must be met by tuning gene expression and enzyme activity [18-20]. Understanding the evolution of the adaptive strategies that assure cell survival in different conditions is, thus, an important goal in Systems Biology [18-22].

In unicellular organisms, adaptive capability is specially important as they lack an internal milieu than could buffer the environmental changes. In this context, the adaptive responses to different stress conditions, (heat shock, oxidative stress, osmotic stress, and other environmental changes), have been extensively investigated using yeast as a biological model [23-26]. In general, such adaptive responses require synthesis of protective proteins (chaperons, trehalose, sphingolipids, etc.), and a fine tuning of the metabolic status to assure an appropriate supply of energy and building blocks for new proteins. The operating principles of the adaptive response to heat shock were investigated under this perspective [18,20]. The increase in trehalose, ATP, and NADPH synthesis are primary requirements for an appropriate response in this conditions. However, these flux constraints cannot fully explain the observed changes. Complementing flux constraints, economy savings in the gene expression changes and constraints for preventing an unnecessary increase of metabolites were shown to be necessary to understand the adaptive response to heat shock. Thus, a combination of constraints on particular metabolic processes, economy issues, fluxes, etc. are required to define the scenario in which natural selection operates [20].

In this paper we develop a new approach that focuses on identifying feasible parametric regions that contain solutions for system parameters so that a set of physiological constraints are satisfied. With that method we will be able of identifying the possible evolutionary solutions that are expected to contain the actual adaptive response. First, we present a global optimization procedure that capitalizes on the properties of a particular class of nonlinear models, the GMA (Generalized Mass Action) models based on the power-law formalism. This method improves the method recently proposed by Polisetty *et al*. [27] and takes advantage of the properties of the power-law formalism as a canonical nonlinear modeling technique [28-30]. Second, we introduce a search strategy that allows identifying the parameter regions containing admissible solutions for the problem. The proposed algorithm is very efficient for realistic problems, although different technical improvements are possible to optimally scale it to large problems. The feasible regions found would represent the landscape in which evolutive solutions are expected. Comparison of our result and actual data allows to discuss the practical usefulness of the proposed approach.

## Methods

### Problem statement

Generally speaking, a metabolic network can be characterized by its processes, internal metabolites, and control (external) variables. Details on the regulatory effects (for instance feed-back inhibition) are important for a complete description. Furthermore, kinetic details are required for computing the response of the system to different inputs and environmental changes. For a system with *p *processes that can contribute to the change in the concentration of the pool of any of the *n *internal metabolites, the basic mathematical representation is(1)

Here, *μ*_*ir *_is a stoichiometric factor that indicates how many molecules of *X*_*i *_are produced or used by the process *v*_*r*_; it is a positive integer if the flux *r *produces *X*_*i *_and it is a negative integer, if the flux *r *depletes the pool of *X*_*i*_.

Each velocity can be represented by different functional forms that may include various parameters. The choice of a particular mathematical form is not trivial and can either facilitate or hinder the task of analyzing the optimal design and the adaptive responses to different environmental changes [31,32]. From all the available formalisms, the so-called *power-law formalism *is one of the most convenient. Details for this choice can be found elsewhere [31]. In this formalism, each velocity is represented as:(2)

In this representation, *X*_*j *_accounts for the concentration of metabolite *j*, *γ*_*r *_is an apparent rate constant for flux *r*, and *f*_*rj *_is the kinetic order of variable *X*_*j *_in reaction *r*. Each kinetic order quantifies the effect of the metabolite *X*_*j *_on flux *r *and corresponds to the local sensitivity of the rate *v*_*r *_to *X*_*j *_evaluated at the operating point indicated by the subscript 0, that is:(3)

If *X*_*j *_has no direct influence on the rate of reaction *r*, the kinetic order is zero. If it directly activates the flux of reaction *r*, the kinetic order is positive. If it inhibits the flux of reaction *r*, then the kinetic order is negative.

Without loss of generality, enzyme levels can be considered embedded in the *γ*_*r *_parameters. This is so because, in most cases, the kinetic-order for the enzyme is 1, as velocities are linearly dependent on the enzyme. Of course, if necessary, enzymes can also be included in the model as control variables. On the other hand, if the model includes dynamic changes in enzymes, for instance through gene regulation, they can also be considered as dependent variables.

Using this representation, a Generalized Mass Action (GMA) model is defined by expressing each velocity in (1) using its power-law form (2) [33]:(4)

Here, we assume that there are *m *≥ 0 control (*external*) variables. Alternatively, an S-system model is obtained by aggregating the different processes in a net process of synthesis  and a net process of degradation  for each metabolite, i.e.:(5)

Using the power-law representation for each aggregated process, we obtain:(6)

In this last formulation, the kinetic orders are called *g *and *h*, and the rate constants *α *and *β *for convenience. The different parameters of models (4) and (6) can be obtained, for instance, via estimation from dynamic data [34,35]. If this is not possible, tentative parameter values can be suggested based on the literature [22,36]. Furthermore, based on their mathematical definition, values that represent those situations of interest can be easily proposed even in absence of specific data. This last possibility allows model analysis and exploration even in cases were little experimental information is available [37,38]. GMA and S-system models are very interesting, as they capture the underlying non-linearities of the system processes and provide a model that is amenable to optimization techniques [39]. Here we will separate two different goals:

1. **Optimization in biotechnological applications**: Given a model that represents the reference state of the system, we are interested in finding the parameter changes (*engineering design*) that optimize a given objective function (usually a flux).

2. **Find feasibility regions in evolution studies**: Given a model that represents the normal metabolic state of a cell, find the admissible changes at the level of enzyme activities that are compatible with a set of physiological and functional effectiveness criteria (*evolutive emergence of design*).

## Results and discussion

### Optimization approach

#### Motivation of the optimization approach

Optimization of biological processes is a very important goal in biotechnological applications [40-44]. In general, the main purpose is finding the appropriate changes in enzyme levels, mainly through changes in gene expression, so that a given objective function is optimized. This can be a flux, for example in a production process, a metabolite concentration, or any other objective function. Developing appropriate optimization techniques is fundamental for defining a practical metabolic engineering approach [44]. The intrinsic complexity of the target systems and the non-linearities involved in the underlying processes makes optimization a difficult task in this field. The use of models based on approximated representations such as the power-law models, either in their S-system or in GMA form, is a promising alternative [19,45].

Optimization based on nonlinear models defined with the power-law formalism was first proposed by Voit [45]. By using the S-system model strategy, a transformation to logarithmic coordinates allows using linear optimization for solving a number of biotechnological problems [45-47]. However, when the problem is represented by a GMA model as in (4), this technique cannot be directly applied. To overcome this problem, methods for optimizing models based on this particular form have been developed [27,39,47]. Here, we shall present a new method that is closely related with the method suggested by Polisetty et al. [27]. Our first goal is to develop an efficient global optimization method for GMA models. Besides its own interest for metabolic engineering tasks, this is a requirement for developing the feasibility approach that is the ultimate goal of this work.

#### Optimization method

In practical biotechnological applications, we are interested in obtaining the best set of changes in enzyme levels (that is *γ*_*r *_in GMA models), so that a given goal is attained (for instance maximize a flux) and a set of constraints are satisfied (for instance: metabolite levels do not increase over a given threshold, some reactions do not reduce the fluxes under given values, changes in enzymes do not go beyond a realistic maximum, etc.)

The optimization task can be posed as an standard optimization problem that aims to identify the specific values of *v*_*r*_, *γ*_*r *_and *X*_*j *_that minimize a given predefined criterion while satisfying at the same time a set of biological constraints. In what follows, we consider an standard optimization problem in which the objective function is minimized. Maximization problems can be easily reformulated into minimization problems by changing the sign of the objective function. At this point, kinetic-orders are regarded as fixed parameters. These parameters represent intrinsic kinetic properties of the involved processes. Optimization of their values is possible, although the method should be further adapted to deal with that case and assure a global optimum. We shall consider this possibility in the future.

The optimization task is, thus, formulated as a nonlinear programming (NLP) problem of the following form:(7)

where **ONLP(S) **denotes the optimal objective value over *S*. The set *S *is called the set of feasible solutions, and contains all the values of *v*_*r*_, *γ*_*r *_and *X*_*j *_that satisfy a set of constraints on the GMA equations. In matrix notation:(8)

In this representation, *v, γ, X *denote the (column) vectors of continuous variables;  is the objective function; and  represents the equations that define the feasible set. In general, *S *may also include additional equations others than those involved in the GMA representation to reflect specific biological conditions. Problem **ONLP(S) **can also be expressed in a more detailed way as follows:

Here the feasible set *S *includes the steady-state equations as a basic constraint. Thus, optimization is run to find a steady-state solution that optimizes the objective function and that fulfills the considered constraints. As commented before, the optimization model may also incorporate other equations that impose certain conditions on the values of the variables *v*_*r*_, *γ*_*r*_, *X*_*j*_. For instance, we can consider a constraint *X*_1 _+ *X*_2 _+ *X*_6 _≤ *k *that forces the summation of certain concentrations of metabolites to be lower than a desired upper limit *k*. This second type of constraints may represent specific physiological properties that the solution sought should satisfy.

Hence, the objective of the NLP model defined above is to find the solution that simultaneously minimizes the function *U*(·) and satisfies the equations (physiological constraints) defined upon the biological system. There are currently many strategies available to solve NLP problems like **ONLP**. These methods are typically implemented in software packages that allow the solution of models with thousands of variables and constraints (see [48]). Unfortunately, there is a particular difficulty in solving the NLP defined by **ONLP**, which stems from the fact that its feasible space is nonconvex. This nonconvex structure is given by the nonlinear equality constraints that define the velocity terms. In nonconvex models, standard NLP techniques are not guaranteed to converge to the global optimum and yield solutions that must be regarded as locally optimal. In the context of performing a biological analysis, this limitation constitutes a major shortcoming, since it can lead to wrong conclusions.

To overcome this drawback, it is necessary to resort to a specific type of mathematical methods known as global optimization techniques (for a detailed review see [49]), which are able to assure the optimality of the solutions found within a desired tolerance. Although such strategies can deal with any type of non-convexities, they tend to be computationally expensive, which hampers their practical implementation. A possible way to reduce their computational burden consists of developing tailored methods that exploit the mathematical structure of the non-convexities of the model.

In this paper, following this general idea, a novel deterministic global optimization method inspired on the works of Bergamini and co-workers [50-52] is presented to solve **ONLP **to global optimality. The method introduced relies on hierarchically decomposing the original problem into two levels, an upper level master problem **CMILP **an a lower level slave problem **RNLP**, between which the algorithm iterates until a termination criterion is satisfied.

The master level of our algorithm entails the solution of a mixed-integer linear programming (MILP) problem, which is a relaxation of **ONLP **(i.e., it rigorously overestimates the feasible region of **ONLP**), and therefore predicts a valid lower bound on its global optimum. Such a model is constructed by replacing the non-linearities in **ONLP **by valid over and under estimators. Particularly, in our method, supporting hyper-planes and piecewise linear functions are used to approximate the original search space of **ONLP**. In the lower level, the original problem is locally optimized in a reduced search space, thus yielding an upper bound on its global solution. The upper and lower level problems are solved iteratively until the bounds converge. For clarity, technical details of the main features of the proposed algorithm are commented in the Appendix section.

#### Algorithmic Steps

**Figure 1:**
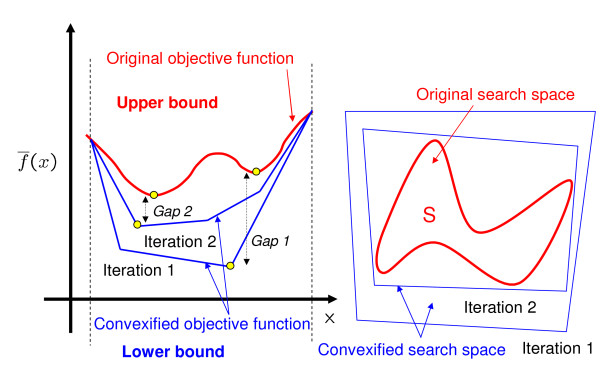
**Optimization algorithm based on outer approximation**. The original nonconvex problem (upper bound) is depicted in red, whereas the convexified problems (lower bound) are shown in blue color. The master problem is used to provide lower bounds to the global optimum and to initialize the slave problem. The bounds tend to converge as iterations proceed.

Figure 1 shows, in an illustrative way, how the algorithm works. At each iteration, the master problem is solved to provide a lower bound on the global solution to **ONLP**. In the master problem, both, the objective function and search space of the original problem are convexified in order to avoid local optima. This is done by introducing auxiliary binary variables that allow to linearize the nonlinear terms. This leads to a reformulated master problem that can be solved to global optimality by standard optimization methods. As discussed on the Appendix, the convexification of the original problem takes advantage of the mathematical structure of the GMA representation. Note, however, that the solution found by the master problem does not necessarily have to satisfy all the constraints in **ONLP**, since some of them may have been relaxed to reformulate the nonconvexities.

Hence, the prediction made by the upper level must be checked in the lower level, so the solution is guaranteed to be feasible in the original search space. Specifically, in our algorithm, the solution of the master model is used as a starting point in the lower level problem, which is solved in a reduced search space that is defined according to the output of the master level. The lower level solution is then employed to tighten the search space of the master problem. As a result, the new modified master problem predicts better lower bounds that are closer to the global solution. Furthermore, tightening the search space of the master problem also improves the quality of both, the starting points and reduced search spaces passed to the lower level. This can be observed in Figure 1, which shows how the envelopes employed in the master problem become tighter as iterations proceed. As a result, the upper and lower bounds tend to approximate and finally converge within the desired optimality gap.

**Figure 2:**
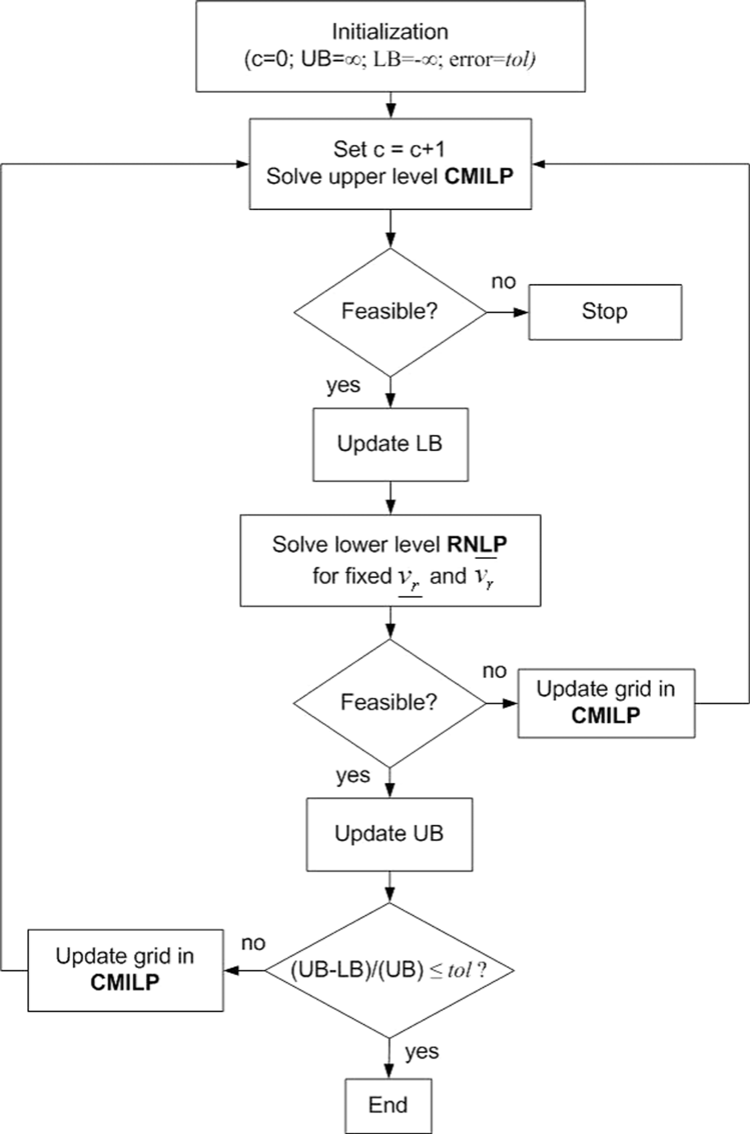
**Flowchart of the optimization algorithm based on outer approximation**.

The detailed steps of the proposed decomposition strategy are as follows (see Figure 2):

1. Set iteration count *c *= 0, upper bound *UB *= ∞, lower bound *LB *= -∞, and tolerance error = *tol*.

2. Set *c *= *c *+ 1.

Solve the MILP master problem** CMILP**:

• If problem **CMILP **is infeasible, then stop. There is no feasible solution to the problem.

• Otherwise, update the current lower bound as follows: , where *LB*^*c *^represents the objective function value associated with the optimal solution of **CMILP **in iteration *c*.

3. For fixed lower and upper bounds on the velocity terms (i.e.,  and , respectively), solve the lower level problem **RNLP **to obtain an upper bound on the solution of **ONLP**.

• If problem **RNLP **is infeasible, then update the grid in **CMILP **and go to step 2.

• Otherwise, update the current upper bound as follows: , where *UB*^*c *^represents the objective function value associated with the optimal solution of **RNLP **in iteration *c*.

4. Check the convergence criteria:

• If , then stop. The solution corresponding to *UB *(i.e., the solution of model **RNLP **in the iteration with minimum objective function value) satisfies the finalization criterion (i.e., it can be regarded as optimal within the predefined optimality gap).

• Otherwise, update the grid in **CMILP **and go to step 2.

#### Optimization of the anaerobic fermentation pathway in *Saccharomyces cerevisiae*

As an illustrative example of the proposed technique, we use the anaerobic fermentation pathway in *Saccharomyces cerevisiae *presented in Polisetty *et al*. (Case study 1 in [27]) as a benchmark problem for optimization. The reader is referred to the original publication for details (Figures 1, 2 in [27]). The model can be found in the Additional File 1.

The algorithm was implemented in GAMS interfacing with CPLEX 9.0 and SNOPT 6.2 as main MILP/NLP optimization packages, respectively, on an Intel 1.2 GHz machine. The upper level of the algorithm was constructed using 50 supporting hyper-planes and 9 piece-wise terms. These upper and lower estimators were updated during the execution of the algorithm by defining new linearizations and terms of the piece-wise approximation in the middle points of the active subintervals in the solution of the master problems. The tolerance error (i.e., optimality gap) was set to 0.2%.

In the upper part of Table 1 we present the optimal solution for three different strategies. As in [27], we limit the possible changes to 5-fold. In those conditions, an optimum rate of 157.59 mM min^-1 ^is found when all enzymes can be changed. This is a theoretical situation that, in practice, can be difficult, if not impossible, to implement. In practice, enzyme manipulation may be restricted to only few enzymes. Thus, we explored a more realistic situation and obtain the optimal changes that would be necessary for maximizing ethanol production if only two different enzymes were to be manipulated at once. As in [27], our method finds that a five-fold increase in glucose transporters and a 2.85 fold-change in phosphofructokinase activity is the best combination if only two enzymes can be changed. In this situation a final rate of 103.66 mM min^-1 ^can be attained. Thus, a realistic strategy may reach about a 66% of the best theoretical solution in that case. In the extreme case that only one enzyme can be manipulated, the best solution is found by increasing glucose intake. This is not surprising as glucose intake is a bottleneck for obtaining a final increase in ethanol production. As illustration of the method performance, we also include in the lower part of Table 1 the optimal solution for all the possible combinations in which glucose intake is increased and a second enzyme is manipulated. In that case, any other strategy of increasing GLK and other enzyme produces a much lower increase in ethanol production that the optimal pair GLK and PFK. All those results are the same as in [27], showing that our method reaches appropriate results.

**Table 1:** Optimization of ethanol production.

Modified reaction numbers	(*X*_*i*_)_*opt*_/(*X*_*i*_)_*nom*_	LB (NLP)	UB (MILP)	Iterations	Total time (s)
HXT	[5]	72.68	72.77	4	2.05
HXT, PFK	[5,2.85]	103.66	103.86	3	2.64
All	[5,0.9,5,0.2,5,0.2,5,5]	157.59	157.70	4	0.89

HXT, GLK	[5,0.75]	72.68	72.77	4	1.32
HXT, PFK	[5,2.85]	103.66	103.86	3	0.57
HXT, GAPD	[5, 5]	73.18	73.31	4	1.22
HXT, PYK	[5,0.63]	72.68	72.77	4	1.25
HXT, TPS+GOL	[5,0.2]	73.41	73.54	4	1.24
HXT, GLY	[5, 5]	73.41	73.54	4	1.22
HXT, ATPase	[5, 5]	87.77	87.84	4	1.24

Best optimization strategies for increasing the rate of ethanol production in the model discussed as case study 1 in [27]. LB: Lower bound, UB: Upper bound. Reaction numbers refer to the mathematical model used in [27]. HXT: Glucose uptake, GLK: Hexokinase, PFK: Phosphofructokinase, GAPD: Glyceraldehide-3-phosphate dehydrogenase, PYK: Pyruvate kinase, TPS+GOL: Polysaccharide production (glycogen + tre-halose), GLY: Glycerol production.

In Table 1, the main computational details of the algorithm, which include the values of the NLP and MILP solved in the last iteration, the total number of iterations and the total solution time are also provided. From a technical point of view, it is worth to indicate that our method produces much tighter upper bounds than those reported in [27] (compare Table 1 with Table III in [27]). For instance, for the case when two enzymes are accessible to manipulation, our method yields an upper bound of 72.68 mM min^-1^, whereas in [27] it is 126.11 mM min^-1^. This means that our method assures convergence to the global minimum within an optimality gap of 0.19%, whereas in the solution reported in [27] this gap is 22%. Similar results can be observed in the remaining optimizations. This advantage can be important for an appropriate scaling of the method for more complex problems. Note that the total time indicated in Table 1 refers to the exploration of all the combinations within each strategy. Thus, the one-enzyme optimization (the case when only one enzymes can be changed) takes 2.04 CPU seconds. The case of finding the maximum when two enzymes can be changed takes 2.64 seconds, whereas the examples in which all enzymes can be modified is solved in 0.89 CPU seconds. Note that at a first glance, one would expect that the problem in which all the enzyme changes are allowed would take more CPU time than those in which only a subset of changes are permitted. These results, which are due to some implementation details of the algorithm, can be found in small problems (i.e., around 1 second of CPU time) but are not likely to appear in bigger problems in which the CPU time is indeed dominated by the complexity of the model rather than by the implementation details.

#### Optimal adaptive response of yeast to heat shock

As a motivation for the feasibility approach that we shall develop in the next section, we shall obtain the optimal enzyme activity changes that would correspond to different goals in the adaptive response of yeast to heat shock. This problem was first analyzed by intensive computation by Vilaprinyo et al. [20]. By using a GMA model of the major metabolic pathways in yeast central metabolism, the goal is to identify which changes at the level of enzyme activities are more likely to produce a desired adaptive response. This response is defined by a set of physiological changes that can be considered as necessary for adaptation. In the model we include glycolysis, synthesis of trehalose and glycerol, and the branching from glycolysis to the pentose phosphate pathway. There are nine enzymes that can be changed, and the target fluxes are those of trehalose, ATP, and NADPH synthesis. Model details can be obtained from [18,20] (see also Table 1 for nomenclature, and the Additional File 1 for the model equations and physiological constraints).

We shall obtain the optimal changes that optimize different goals: (i) Maximization of the rate of trehalose synthesis, (ii) Maximization of ATP synthesis, (iii) Maximization of NADPH synthesis, and (iv) Minimization of cost related to changing gene expression. We consider two different scenarios. First, we run the optimization procedure without physiological constraints. Second, we consider the constraints in Table 2 (see also Additional File 1). The implementation details of the algorithm are the same as in the previous example. Results are shown in Table 3. The table also provides the computational details of the algorithm.

**Table 2:** Physiological constraints that shape the admissible adaptive response to heat shock in yeast.

Metabolites (mM)	Fluxes (mM min^-1^)	Other
Glucose < 0.04	*V*_*TRE*_> 0.03	Cost < 12.06
Glucose-6-P < 20.22	*V*_*NADPH*_> 3.53	
8.64 < Fructose-1,6-P < 22.86	*V*_*ATP*_> 180.6	
Phosphoenol piruvate < 0.01	*V*_*GLY*_> 0.39	
ATP < 6.77	*ψ *< 28.1	

See details in the work of Vilaprinyo *et al*. [20].

**Table 3:** Optimization results for the model of heat shock response in yeast

Objective function	V_*TRE*_	V_*NADPH*_	V_*ATP*_	Cost	V_*TRE*_	V_*NADPH*_	V_*ATP*_	Cost
Goal (Maximize/Minimize)	Max	Max	Max	Min	Max	Max	Max	Min
**Constrains in **[20]	No	No	No	No	Yes	Yes	Yes	Yes
								
Enzymes (Chanfe-fold)								
HTX	20.00	20.00	20.00	1.00	10.66	12.77	19.21	4.52
GLK	0.50	3.00	0.50	1.00	4.58	5.42	8.15	2.42
PFK	0.25	0.25	20.00	1.00	1.00	1.00	2.34	1.00
TDH	0.25	0.25	0.25	1.00	5.11	2.10	4.92	1.46
PYK	20.00	20.00	20.00	1.00	10.54	5.54	11.78	3.07
TPS+GOL	20.00	0.25	0.25	1.00	20.00	2.34	2.34	4.93
GADP	0.25	20.00	0.25	1.00	1.71	20.00	1.71	1.79
Glycerol production	0.25	0.25	0.25	1.00	1.00	1.00	1.00	1.00
ATPase	0.25	0.25	20.00	1.00	1.81	0.62	2.04	1.00
								
**Metabolites (mM)**								

Glucose (internal)	0.75	0.08	22.67	0.06	0.04	0.04	0.04	0.04
Glucose-6-P	278.04	160.28	0.02	0.23	20.22	20.22	20.22	7.95
Fructose-1,6-P	669.48	510.46	164486.70	14.36	22.86	22.86	22.86	22.86
PEP	0.00	4.9 × 10^-4^	1.7 × 10^-3^	3.5 × 10^-3^	0.01	8.3 × 10^-3^	0.01	0.01
ATP	20.54	13.10	6.6 × 10^-5^	0.03	2.47	6.77	6.77	3.63
								
**Fluxes (mM min^-1^)**								

V_*TRE*_	2.08	0.02	1.3 × 10^-5^	3.6 × 10^-4^	0.26	0.03	0.03	0.03
V_*NADPH*_	0.59	46.21	0.36	1.64	3.54	41.44	3.54	3.53
V_*ATP*_	154.90	247.16	1755.86	49.05	620.09	281.06	658.84	180.47
V_*GLY*_	0.00	0.01	877.66	22.22	1.00	0.40	0.44	0.73
Ψ	5.00	0.06	5.00	1.00	20.00	2.34	5.48	4.93
Cost	16.61	17.02	18.22	0.00	12.00	11.02	12.06	6.07
								
**Computational details**								

Iterations	4	4	4	1	1	4	5	3
LB (NLP)	2.08	46.21	1755.86	0.00	0.26	41.44	658.84	6.07
UB (MILP)	2.09	46.29	1757.87	0.00	0.26	41.48	659.74	6.06
Total time (s)	1.06	1.83	2.54	0.43	0.22	0.97	2.12	1.19

Constraints correspond to those in Table 2. For details on the model and on the meaning of Ψ and Cost the reader is referred to [20] and to Additional File 1.

Trehalose synthesis can be increased up to 2.08 mM min^-1 ^by increasing the activity of HTX, PYK, and TPS+GOL, while decreasing most of the other activities. In doing that, two of the involved metabolites (G6P and F16P) accumulates. Furthermore, synthesis of glycerol is reduced to zero and synthesis of NADPH is decreased. This optimal solution cannot be considered a good adaptive solution as it compromises the cellular inner milieu by accumulating metabolites and challenges other processes by decreasing some critical fluxes. In that sense, this solution optimizes a metabolic goal (*v*_*TRE*_) but does not match other important physiological constraints. A similar result is obtained when the goal is to maximize the synthesis of NADPH or ATP. Particularly striking is the unrealistic concentration of F16P that is obtained in the optimal strategy for maximizing ATP synthesis. In the case of minimization of Cost, as no further constraints are imposed, we find the trivial solution that corresponds to maintain the basal conditions.

Considering the set of constraints identified by Vilaprinyo et al. (Table 2), we run again the optimization procedure for each of the four objective functions. In all the cases, an optimal solution compatible with the imposed constraints is obtained. For instance, the maximum rate of trehalose synthesis that is compatible with the considered constraints is 0.26 mM min^-1^. This is well below the maximum obtained without constraints, but now the solution is reasonable in the terms imposed by the physiological constraints. Similar results are obtained for the other objective functions. We also obtain the solution that minimizes the overall cost. In that case, cost can be lowered about a 50% with respect the other cases. Minimization of cost undertakes values of the fluxes that are lower than in the optimal solutions for the other objective functions. From the implementation point of view, it is interesting to notice that in all the cases the algorithm was able to provide near optimal solutions (with an optimality gap of 0.2%) in few CPU seconds. Similarly, as in the previous example, the master problem provided very tight relaxations, which led to few iterations.

### Feasibility approach

Optimization techniques seek finding the best strategy for changing control variables so that the system can reach a given goal. Thus, such methods are important in biotechnology and metabolic engineering where the scientist fixes the goal and searches for the best strategy in changing the underlying processes. The situation is different if we analyze the evolution of natural systems. As discussed above, natural systems are (in some sense) optimized by the evolutive process by natural selection that acts as a purifying process. Although the exact contribution of this mechanism is still discussed [53], it is generally accepted as one of the basic driving forces in evolution. From this perspective, different criteria for functional effectiveness have been used for investigating the emergence of design principles in cellular systems [5,16,17].

This part of the paper introduces a method that aims at providing an approximate characterization of the feasible space of a biological problem rather than identifying a single optimal solution. The tool presented is intended to provide valuable insights on the set of changes in enzyme activities that would be required for adaptation to an environmental challenge.

The approach introduced is based on the NLP formulation defined in **ONLP **and exploits the specific structure of the GMA representation. The use of this particular representation allows to perform the feasibility study by slightly modifying the algorithm described in the previous section. Specifically, our strategy relies on solving the original problem **ONLP **iteratively over a reduced search space. At each iteration the method identifies a region that contains a feasible solution to the problem. This region is then removed from the search space, and the optimization problem is resolved in the reduced domain. The algorithm proceeds in this manner until there is no feasible solution in the remaining regions of the search space.

The overall method, which relies on the global optimization approach introduced before, comprises two different levels. At the upper level, a master problem is solved to identify a region that may contain a feasible solution to **ONLP**. At the lower level, the prediction made by the master problem is checked by solving the original problem in a reduced search space. If a feasible solution is found, then integer cuts are added to the master problem in order to exclude the region containing such a feasible point. Otherwise, the master model is updated by refining its grid, until either a feasible solution is obtained in the lower level or an infeasibility is detected in the higher level problem. The main features of the algorithm are outlined next, whereas more technical details can be found in the Appendix.

#### Mathematical representation

We define a set of disjoint sets  ( for all *q *≠ *q'*) such that their union contains the feasible space *S *of **ONLP **(). In our work, for the sake of simplicity but without loss of generality, we assume that each of these regions  is a hyper-rectangle described by a set of linear inequalities that impose lower and upper limits on the apparent rate constants *γ*^*q*^(*γ*^*q*^ and , respectively). Thus, we have:(9)

This assumption implies that the feasibility analysis will be performed on the values of the apparent rate constants *γ*, although in general it could also account for other variables. In this notation, the values of the *r *components of *γ*^*q*^ and , which are denoted by  and , respectively, correspond to the limits of the subintervals obtained by partitioning the original domain of each single variable *γ*_*r *_[0, ∞] into *T *subintervals. It follows that *T*^*r *^= *Q*. In each hyper-rectangle , the component *r *of the vector *γ *must fall into a specific subinterval .

**Figure 3:**
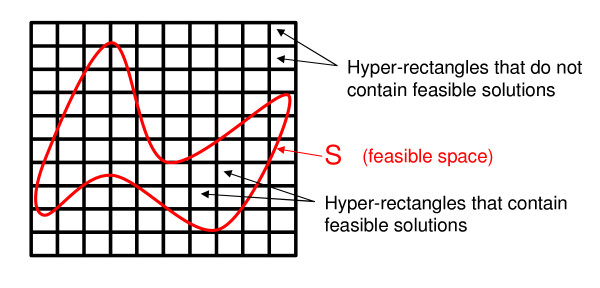
**Definition of hyper-rectangles**. The overall domain is divided into a set of hyper-rectangles that contain the original nonconvex search space of the model. Each hyper-rectangle considered in the analysis can either contain at least one feasible solution or be empty. Hyper-rectangles dissected by the feasible space (i.e., those placed in its borders) contain feasible and unfeasible parts.

In mathematical terms, the feasibility analysis consists of identifying, from the aforementioned set of hyper-rectangles, those that contain feasible solutions to **ONLP **and those in which no feasible point exists (see Figure 3). In other words, we aim at determining whether the intersection *S *∩  is empty (*S *∩  = ∅) or contains at least one feasible point (*S *∩  ≠ ∅).

#### Algorithmic Steps

The algorithm relies on solving two different problems, a modified master problem **CFMILP **and a modified slave problem **RFNLP**, between which the algorithm iterates. Model **CFMILP **is obtained from **CMILP **by adding a set of auxiliary equations that define the set of hyper-rectangles on which the search is conducted. Furthermore, it also incorporates a set of integer cuts that exclude from the search space those hyper-rectangles that have been explored so far. Similarly, model **RFNLP **is derived from **RNLP **by imposing certain lower and upper bounds on the values of the apparent rate constants *γ*. These bounds correspond to the limits of the hyper-rectangle that contains the solution predicted by the master problem, which may or may not be feasible in the search space of the original problem **ONLP**.

Model **CFMILP **is a relaxation of **ONLP **and therefore predicts a valid lower bound on its solution. Furthermore, if **CFMILP **is unfeasible, it follows that **ONLP **(and also **RFNLP**) are unfeasible in any hyper-rectangle contained in the search space of the master problem (i.e., any hyper-rectangle that has not yet been removed from the search space). In our approach, this property is indeed exploited to terminate the algorithm.

**Figure 4:**
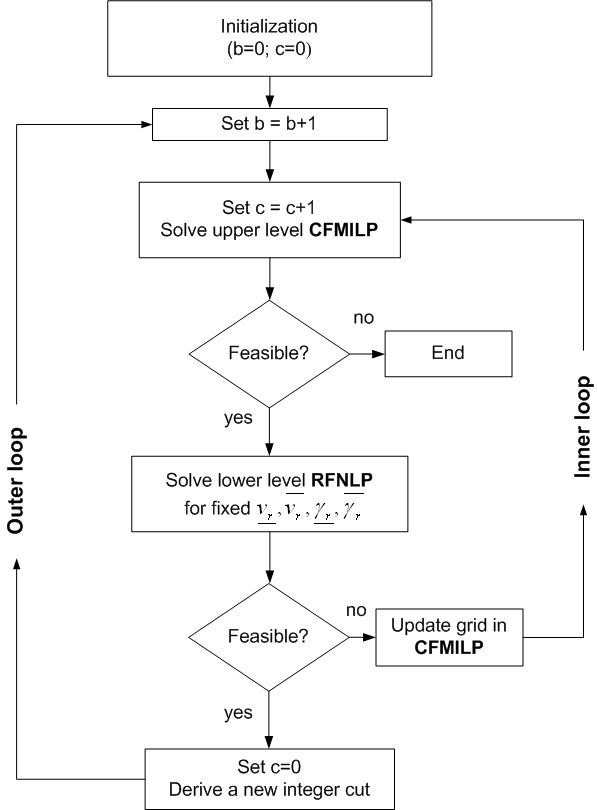
**Flowchart of the algorithm for identifying feasible parameter regions**.

The detailed steps of the proposed algorithm are as follows (see Figure 4):

1. Set outer iteration count *b *= 0, inner iteration count *c *= 0, upper bound *UB *= ∞, lower bound *LB *= -∞.

2. Set *b *= *b *+ 1.

3. Set *c *= *c *+ 1.

Solve the MILP master problem **CFMILP**:

• If problem **CFMILP **is infeasible, then stop. There is no feasible solution to the problem in the current search space.

• Otherwise, for fixed lower and upper bounds on the apparent rate constants (, ) and on the velocity terms (, ) solve the lower level problem **RFNLP**.

- If problem **RFNLP **is infeasible, then update the grid in **CFMILP **and go to step 3.

- Otherwise, derive a new integer cut, set *c *= 0 and go to step 2.

#### Feasible enzyme activity patterns in the adaptive response of yeast to heat shock stress

Using the same model as in the second optimization example, we have investigated the feasibility regions for changing enzyme activities in yeast metabolism so that specific physiological constraints are met. For simplicity in representing the results of the analysis, we shall present 2-D plots in which the feasible regions for the changes in two enzymes are shown. It is important to indicate that in each case all the enzymes are considered in the feasibility analysis, although only the corresponding region for the two selected enzymes is shown.

According to that, the hyper-rectangles for the feasibility analysis are defined on the domain of the selected enzymes, say for instance PFK and TDH. For practical reasons, we consider *γ*_*r *_= *k*_*r*_*γ*_*r*0_. Here, *γ*_*r*0 _represents the basal value and *k*_*r *_the change-fold over the basal value. We shall use the values of *k*_*r *_that correspond to the relative change of that enzyme with respect the basal activity. In this paper, we consider that enzyme activities are changed only by changing the amount of enzyme. Of course, activity changes due to other reasons, such as temperature dependency, could also be considered. In such cases, the cost component would be much lower. In either case, the resulting changes in activity would affect metabolite concentrations and fluxes. Those changes are considered in other constraints used in this analysis.

As an example, in the case of PFK and TDH 10 equally spaced segments from 0.25 to 20 fold-change are considered in the study. This leads to 100 hyper-rectangles, each of which corresponds to a specific combination of one sub-interval of PFK and another of TDH. On the other hand, no sub-intervals are defined for the remaining enzymes. This implies that in each hyper-rectangle the model is free to choose any values for them. Thus, the method is free for finding the best combination of enzymes that, within the considered hyper-rectangle, optimizes the objective function and meets the constraints. In this example, the synthesis of ATP was regarded as the criterion to be optimized in the master and slave problems of our algorithm. This objective is only used to guide the MILP and NLP subproblems. However, as mentioned before, any other criterion could have been employed, with identical results, since the main goal of our algorithm is to identify feasible regions and not to find optimal solutions. The implementation details of the algorithm are the same as in the previous examples. It is interesting to notice that the total CPU time was 31.81 CPU seconds, which shows the efficiency of the proposed method.

**Figure 5:**
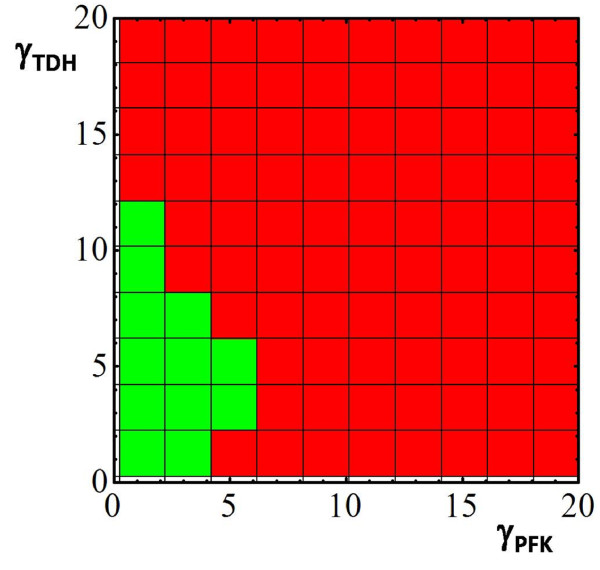
**Feasibility analysis of the adaptive response of yeast to heat shock**. Feasibility analysis of the adaptive response of yeast to heat shock. In each case, all the enzymes are allowed to change, producing a 9-D search space. For simplicity, only the result for PFK and TDH is presented. A 10 × 10 grid of values for the change in activity of PFK and TDH is explored for feasible solutions. Grid values are defined as equally spaced segments from 0.25 to 20. Red squares indicate regions in which no feasible solution is found. Green squares indicate regions in which at least one feasible solution is found.

The results of the feasibility analysis are given in Figure 5. The boxes in green represent hyper-rectangles that contain at least one feasible solution to the problem, whereas those in red have been proved to be unfeasible. As expected, there are numerous enzyme activity patterns that allow meeting these requirements. In concordance with our previous results, the model can find admissible solutions by compensating the activities of PFK and TDH. However, as the increase of PFK reaches its higher limit, admissible solutions can only be found with restricted values of TDH. In the unfeasible region, no compensation on the rest of enzymes produce an admissible solution considering the corresponding values of PFK and TDH.

**Figure 6:**
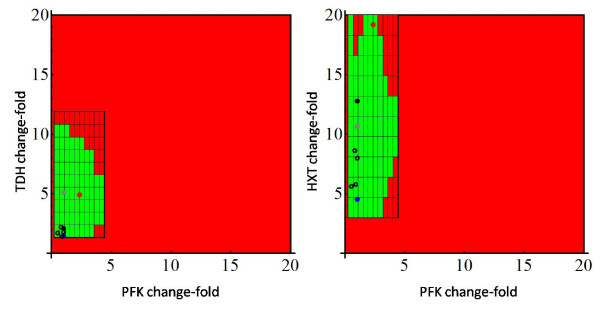
**Feasibility analysis of the adaptive response of yeast to heat shock**. Feasibility analysis of the adaptive response of yeast to heat shock. Once the maximum and minimum regions that contain feasible solutions are found, a refined search is performed. For PFK the parameter set goes from 0.25 to 4.41. For TDH it goes from 1.35 to 11.86. For HXT it goes from 3 to 20. In each case, 10 segments are considered. Color code is as in Figure 5. Red circle: Maximum rate of ATP synthesis; Black circle: Maximum rate of NADPH synthesis; Gray circle: Maximum rate of Trehalose synthesis; Blue circle: Minimum cost; Black empty circles: Experimental observations (Table I in [20]).

Let us note that the search grid employed in the analysis can be easily refined by performing a bound contraction strategy. Specifically, the lower and upper allowable fold-changes for the associated rate constants can be determined by running 4 different optimization problems that account for the maximization/minimization of each single rate constant separately. This strategy reveals, for instance, that the admissible intervals for the rate constant range from 0.25 to 4.41 for the PFK, and from 1.35 to 11.86 for TDH. Figure 6 shows the results obtained when this new grid is considered, assuming again 10 sub-intervals for each enzyme. As can be observed, the advantage of performing the bound contraction is that it allows to discard beforehand regions of the search space that do not contain feasible solutions. Hence, the study concentrates only on the most promising hyper-rectangles (i.e., those in which it is more likely to find a feasible point). The total CPU time in this new case was 420.00 CPU seconds. The existence of a relatively wide region of admissible solutions raises the question of which of the solutions will evolve by natural selection. To shed light on this issue, in Figure 6, we have also plotted the optimal solutions found in Table 3 (filled color points) and some experimental points corresponding to the data bases analyzed by Vilaprinyo et al. (see Table I in [20]). Interestingly, the experimental data, in the case of PFK and TDH, are situated close to the optimal solution that is obtained when the cost is minimized. Roughly, this may be an indication that adaptive response of yeast to heat shock has been shaped to save resources related to over-express enzymes. For illustrative purposes, we also show the results obtained for PFK and HXT. In that case, as the system can compensate larger changes in HXT, results are not all that clear. Iteratively, we could consider all the possible pairs to obtain a global view of the results.

#### Effect of the constraint values

The results obtained in the feasibility analysis shown in the previous section are dependent on the values of the constraints. The values used in that case were obtained in a previous analysis of the model [20] and could be modified in different ways. The interesting advantage of our method is that it allows for exploring the resulting feasible regions in practical terms for large models. Thus, one could consider different values that can be suggested by experts or obtained from other biological considerations. Of course, a sound knowledge of the biological problem is a fundamental basis for this. In any case, comparison of the resulting feasible regions can help in evaluating the considered constraints and may help in identifying those constraints that are more likely responsible for the actual characteristics of the adaptive response. This would require appropriate experimental data for comparing the model predictions with the actual system behavior.

## Conclusion

While the solutions obtained via optimization methods can be realizable in a biotechnological application, provided the required changes can be practically implemented, the optimal changes would seldom be attained in natural systems that have evolved through natural selection. From a general point of view, those systems have evolved so that the metabolic status meets a set of constraints without compromising survival and viability. For instance, adaptive response to a given condition may require to increase a flux over a given limit. However, instead to evolve towards a solution that reaches the maximum possible flux, evolution finds a solution that provides the required increase without compromising other objectives, for instance maintain a low concentration of internal metabolites.

Our feasibility approach has been developed to address this problem. By considering a set of physiological constraints, we search for the feasible parameter regions that allow the system to meet those constraints. We have investigated the utility of this approach in exploring the adaptive response of yeast to heat shock. Our results identify the admissible changes in enzyme activities that can meet the physiological constraints suggested in [20] (see Table 2). Interestingly, the experimental observations are located within the predicted feasibility region and close to the changes that would be required to minimize the cost of overexpressing the different enzymes (Figure 6). Furthermore, the proximity to the optimum for trehalose synthesis also suggest that this is an important requirement for the actual adaptive response.

Although this is not the case in the considered examples, it may happen that two or more unconnected feasible regions may exist for a given problem. That situation would be very interesting from the point of view of discussing the evolution of that adaptive response. In theory, it would mean that solutions in either of the regions could emerge from natural selection. If actual data situates the evolutionary solution in a particular region, then one may ask which were the disadvantages of the other possibilities. Besides a random choice, selection of a given solution among equally admissible alternatives would be a clue of the existence of complementary constraints. For instance, it may be that the evolved solution is better for assuring an appropriate adaptive response to other stress conditions. Also, one should check for differences in dynamic responses as a complementary argument for evaluating the performance of each of the potential solutions.

The practical use of the methodology developed in this work requires a mathematical model that accurately reflects the properties of the system. Furthermore, although detailed models would be desirable, the mathematical complexity makes optimization tasks very difficult on those models. GMA models provide a practical solution and have several advantages that allow an efficient implementation of the optimization procedures. First, automatic generation of models from conceptual schemes is straightforward, which facilitates obtaining a useful mathematical model. Second, a number of techniques exist for fitting those models to dynamic data (see [34] for a review), which is basic for obtaining a numerical model that can be used in optimization procedures. Third, GMA models can incorporate qualitative data, which can help when limited information is available for parameter identification. Finally, the specific structure of the GMA model can be exploited to devise an efficient tailor-made global optimization algorithm. Particularly, in the context of the proposed method, we take advantage of the GMA representation in order to construct master MILPs that provide tight lower bounds on the optimal solution to the original problem. Thus, our method exploits the advantages offered by this kind of models for obtaining a useful implementation of the optimization and feasibility approaches.

In practical problems, once a model has been appropriately identified, parameter uncertainties can be a difficulty for obtaining a reasonable result [54,55]. As the procedure developed here is highly efficient, it should be practically possible to run a sensitivity analysis by screening different parameter sets. This would help in discussing the validity of the obtaining results when parameter variability is an issue.

The proposed method requires a sound knowledge of the biological problem. This is especially important for identifying physiological constraints that can be relevant in limiting the feasibility region. If those constraints are not clearly identified, our method can be used as an exploratory tool for identifying different feasibility regions that would correspond to different sets of physiological constraints. An analysis of the different results could help in identifying those constraints that may be more important in a given scenario. In any case, validation of the feasibility regions obtained would require experimental data. Discrepancies between theoretical results and actual data may help in discarding unreasonable physiological constraints. In the example discussed here, experimental observations agree with the predicted feasibility region. This should be interpreted as an indirect prove that the physiological constraints used are meaningful in that case. However, it is not a prove that these are the only constraints that explain the observed results. An iterative analysis through alternative sets of constraints would be required for completely identifying the most significant ones.

Besides its application to understand the evolution of adaptive responses, our methodology can be useful in exploring health and disease states [56] so that optimal targets for specific treatments via regulation of enzyme activity can be suggested [57]. All these problems must take into account the evaluation of several objective functions [58]. Our approach could simplify this task by finding the optimal solution for each of the various objective functions and by comparing these results within the feasibly region (see for instance Figure 6).

Our method focuses on exploring adaptive responses through changing enzyme activities. It would be interesting to extend the methodology to include kinetic-orders in the optimization. This would allow to explore design principles required for a given network to perform according to specific performance criteria. Also, it would be necessary to adapt the optimization procedures so that dynamic properties, such as stability and response time, could be included as physiological constraints.

## Authors' contributions

AS suggested the need for the feasibility approach and provided the biological problem. GG-G developed both the optimization and the feasibility algorithms and performed the numerical analysis. Both authors evaluated the results, wrote the paper and equally contributed to its final form.

## Appendix

### Upper level master problem for the optimization procedure

The upper level master problem **CMILP **is a relaxation of **ONLP **that is obtained by applying an exponential transformation on some variables of **ONLP **and then replacing the resulting logarithmic terms by valid over and under estimators. We first define the following exponential transformations:(A.1)

where Γ_*r *_and *x*_*j *_are the new transformed variables. With these changes, equation A.2 translates into:(A.3)

A logarithmic transformation is next applied on equation A.3 to obtain:(A.4)

This equality constraint can be replaced by the following inequalities:(A.5)

**Figure 7:**
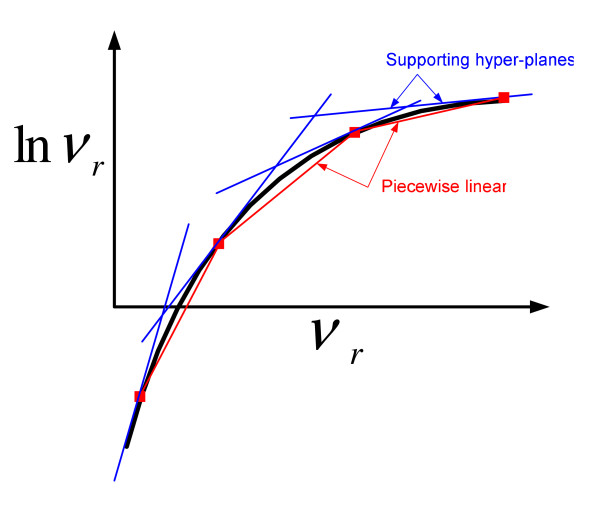
**Approximation of the logarithmic terms**. Approximation of the logarithmic terms via piecewise linear functions (in red color) and supporting hyper-planes (in blue color).

The concave univariate terms appearing on the left hand side of equations A.5 and A.6 are next replaced by valid under and over estimators, respectively. Specifically, in this work we employ piecewise linear functions and supporting hyper-planes to under and over estimate the logarithmic terms, respectively (see Figure 7).

To derive the piecewise linear functions, we consider a partition of the domain [, ], determined by grid points , with  = ,  and  for *h *= 1, ... *H*. The piecewise under estimator can be formulated as a disjunction with *H *terms (see [50]):

The mixed-integer formulation based on the convex hull reformulation [59] is as follows:(A.7)

Then, by combining A.5 and A.8, we get:(A.12)

On the other hand, the logarithmic term in constraint A.6 is approximated by a linear outer-approximation. This is accomplished by adding supporting hyper-planes, which are obtained by performing first order linearizations at a set of *l *points. Equation A.6 is therefore rewritten as follows:(A.13)

Since the logarithmic function is concave, these hyper-planes strictly over estimate its value and thus do not chop off any feasible solution of the original model **ONLP**. The overall master problem can therefore be expressed as follows:

Model **CMILP **takes the form of a mixed-integer linear programming (MILP) problem. This type of model can be efficiently solved via standard branch and bound techniques [60].

Note that the master problem can also be expressed as follows:(A.14)

where the set *RS *is a relaxation of *R *(i.e., contains *R*) and it is defined as follows:(A.15)

where  is the reformulated objective function; and  represents the set of reformulated equations that define the feasible set, which includes the auxiliary constraints that define the lower and upper estimators of the logarithmic terms.

### Lower level slave problem for the optimization procedure

The lower level of the algorithm is represented by a reduced NLP (model **RNLP**), which is obtained from the original nonconvex formulation **ONLP **by imposing lower and upper bounds on the values of the velocity terms.

The solution of such a model provides an upper bound on the objective function of **ONLP**. The master and slave problems are solved iteratively until the upper and lower bounds converge. Note that in **RNLP**, the lower and upper limits of the intervals within which the values of *v*_*r *_must fall (i.e.,  and ) are given by the master problem **CMILP**. Specifically, these bounds correspond to the limits of the intervals that are active in the master problem. Then, if  denotes the solution of the master problem, in which the *h *term of the disjunction that approximates *v*_*r *_is active, then we have that  and, hence, .

#### Remarks

• The upper and lower estimators are only required to replace those velocity terms that appear in equations with more than two terms. On the other hand, equations containing only two of the remaining velocity terms can be written as follows:(A.16)

 where *TE *represents the set of those metabolites whose concentration is described by only two velocity terms, and *TJ*(*i*) represents the set of velocity pairs (*j, j'*) associated with each metabolite *i *in *TE*. Note that in equation A.16, *μ*_*ir *_and *μ*_*ir' *_have opposite signs, so it is possible to perform a logarithmic transformation on both sides of the equation:(A.17)

By combining equations A.4 and A.17, we finally get:(A.18)

which is a linear constraint.

• The grid in problem **CMILP **can be updated in different ways. A possible strategy to perform the updating consists of including in it the middle points of the active subintervals in the solution of the master problem **CMILP**. Therefore, if the solution of **CMILP **is such that  (i.e., interval *h *is active), then the grid corresponding to *v*_*r *_is modified by adding the new point . Alternatively, the grid can be updated by just adding the optimal solution obtained in the lower level problem **RNLP**.

• In each iteration, additional hyper-planes can be added in the master problem in a similar way as it is done with the grid updating. Thus, the logarithmic terms can be linearized either at the middle points of the active subintervals or at the optimal values obtained in the lower level problem **RNLP**.

• The approach presented can easily handle the case in which lower and upper bounds are imposed on the apparent rate constants *γ*_*r *_and/or the concentrations of metabolites *X*_*j*_. These conditions can be expressed via the following constraints:(A.19)

which can easily be converted into the following linear inequalities:(A.21)

• The approach presented also allows to fix upper bounds on the summation of *γ*_*r *_and *X*_*j*_. This can be accomplished by adding the following inequalities:(A.23)

Here, *SG *and *SX *denote the upper bounds on the summations of *γ*_*r *_and *X*_*j*_, respectively. These inequalities can be equivalently written as follows:(A.25)

Constraints A.25 and A.26 are convex, and hence can be linearized in a similar way as was done with equation A.6. Note, however, that the definition of lower bounds on the summation of γ_*r *_and *X*_*j *_leads to nonconvex terms. In this latter case, additional piecewise estimators are required to preserve the convexity of the model.

• Let us finally note that different types of piecewise functions could be applied in the master problem [52].

### Modified master problem for the feasibility method

The problem of identifying if the variable γ_*r *_falls into the subinterval  can be formulated as a disjunction with *T *terms:(A.27)

**Figure 8:**
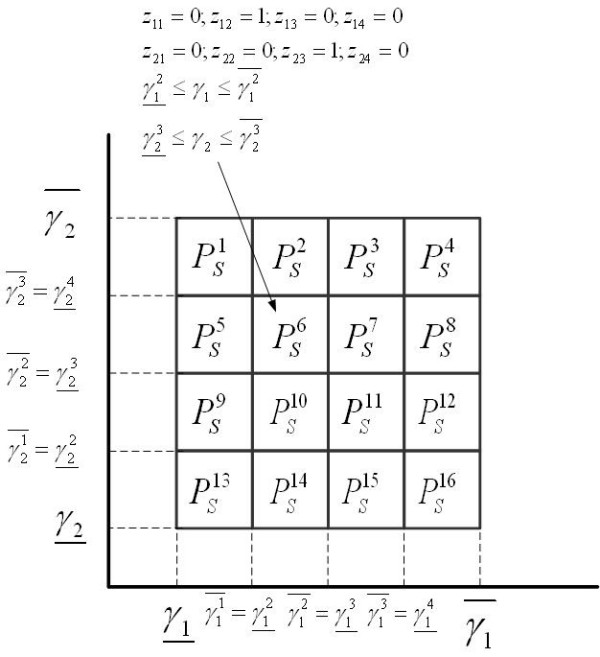
**Feasibility analysis: hyper-rectangles**. The hyper-rectangles are defined by imposing lower and upper bounds on the values of the apparent rate constants. The membership of a solution to a specific hyper-rectangle is defined by a vector of binary variables that identifies the active sub-intervals in which the solution falls.

The mixed-integer formulation based on the convex hull reformulation [59], is as follows:(A.28)

where  is the auxiliary disaggregated variable and *z*_*rt *_is a new binary variable that takes a value of one if γ_*r *_lies in the subinterval *t*, and it is zero otherwise. Figure 8 shows an illustrative example with *r *= 2 and *t *= 4. Note that the binary variables *z*_*rt *_indicate the membership of a solution to a specific hyper-rectangle. In the example presented, each variable is divided into 4 intervals, which leads to 16 different hyper-rectangles, each of which is represented by a different binary solution z*.

The task of identifying optimal values of *v, γ *and *X *according to a predefined criterion over the search space  can be expressed as a mixed-integer nonlinear programming (MINLP) problem with the following form:

The equations in **FMINLP **define a non convex feasible region. However, it is possible to convexify the model by applying the same algebraic transformations described before. Finally, the reformulated MILP problem can be expressed as follows:

In the context of our algorithm, this master problem is employed to predict whether a feasible solution exists in the search space *SP *or not.

### Modified slave problem for the feasibility method

The modified slave problem is a reduced NLP model **RFNLP **that is obtained from the original nonconvex formulation **ONLP **by imposing lower and upper bounds on the continuous variables *v*_*r *_and *γ*_*r*_.

Note that in model **RFNLP**, the lower and upper bounds imposed to *γ*_*r *_are given by the values of the binary variables *z*_*rt *_in the master problem, whereas the values of  and  correspond to the limits of the active intervals of the linear piecewise approximations in **CFMILP**.

### Integer cuts for the feasibility method

At each iteration, the search space is reduced by removing all the sub-regions (i.e., hyper-rectangles) containing feasible solutions that have been identified so far. This is accomplished by making use of integer cuts, which are mathematically expressed as follows:(A.31)

where  and , with  being the value of the *s *component of the vector of binary variables in the feasible solution identified in the outer iteration *b*. The sets  and  are therefore obtained in each outer iteration *b*, and are employed to derive integer cuts that are added cumulatively to the master problem **CFMILP**.

**Figure 9:**
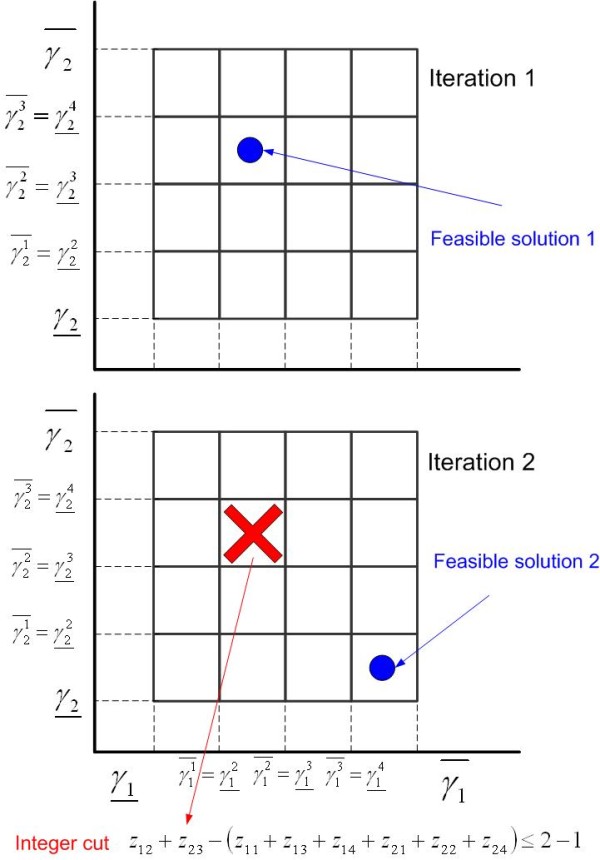
**Feasibility analysis: integer cuts**. Hyper-rectangles containing feasible solutions (blue circles) are removed from the search space in next iterations (red cross) by adding integer cuts. The integer cut guarantees that the combination of active intervals that defines a feasible hyper-rectangle will not be repeated in subsequent iterations.

Figure 9 illustrates how the algorithm removes at each iteration the hyper-rectangles containing feasible solutions identified in previous iterations from the search space. This is accomplished by adding integer cuts, which prevent the master problem from repeating the binary solutions z* that correspond to the feasible hyper-rectangles. This procedure is repeated until an infeasibility is detected in the master problem, which implies that there is not any feasible solution to **ONLP **in the remaining hyper-rectangles of the search space.

#### Remarks

• Note that the algorithm only requires solving the problems to feasibility, i.e., a feasible solution of the problems is sufficient for the algorithm goal. However, by defining a small tolerance error *tol*, the algorithm can also determine the optimal solution in the region *SP *explored at each iteration within the predefined optimality gap.

• The integer cuts in equation A.31 are added cumulatively at each iteration to the upper-level model **CFMILP**, which leads to an increase in its size.

## Supplementary Material

Additional file 1 **Model details and physiological constraints**. Detailed description of the models used in the main text and the physiological constraints considered in the optimization and feasibility examples. Click here for file
