# Supplementary material for: Identifying quantitative operation principles in metabolic pathways: a systematic method for searching feasible enzyme activity patterns leading to cellular adaptive responses
Source: BMC Bioinformatics. 2009 Nov 24;10:386. doi: 10.1186/1471-2105-10-386 (PMC2799421; doi:10.1186/1471-2105-10-386)
Supplement: Additional file 1 — Model details and physiological constraints. Detailed description of the models used in the main text and the physiological constraints considered in the optimization and feasibility examples. [file 1471-2105-10-386-S1.PDF]

Supplementary material to the manuscript

Identifying quantitative operation principles in metabolic pathways: a systematic method for searching feasible enzyme activity patterns leading to cellular adaptive responses.

Gonzalo Guillén-Gosálbez
Albert Sorribas

GMA model for the ethanol optimization example.

This model is taken from (Curto, Sorribas, & Cascante, 1995). This is the same model used in (Polisetty, Voit, & Gatzke, 2006) for the optimization example. The scheme of the model is:

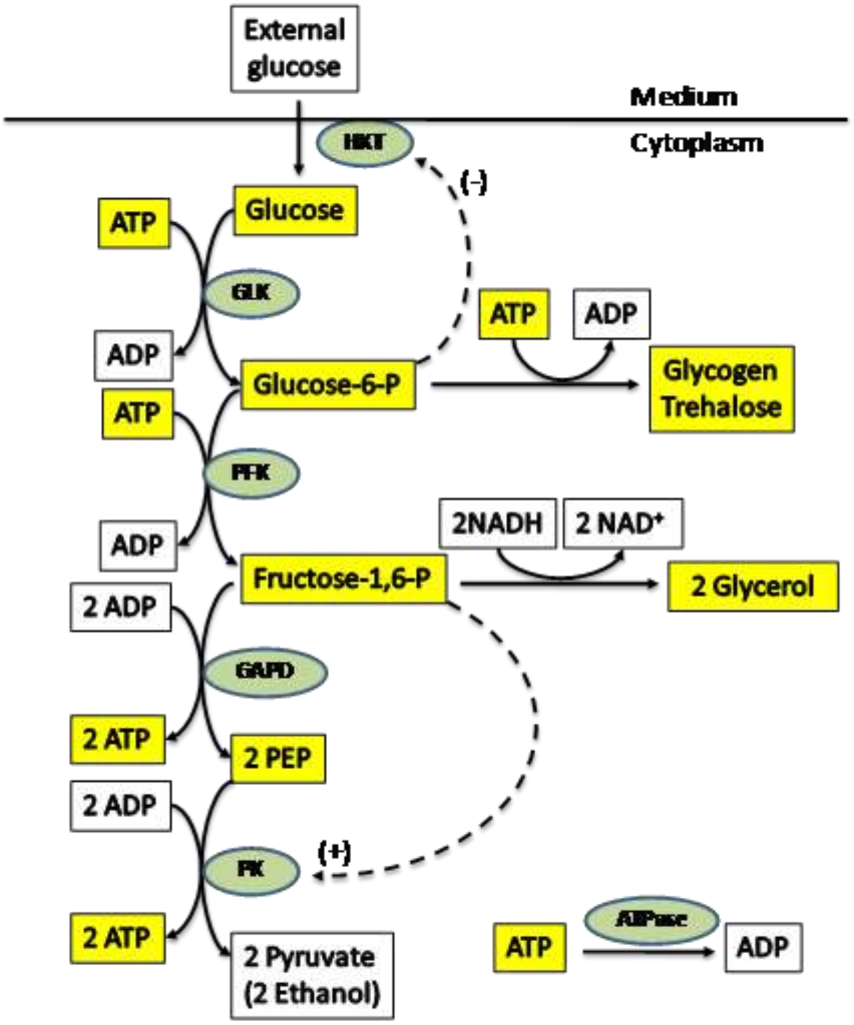

Internal Variables

Symbol	Name	Basal concentration (mM)
X_1	Internal glucose	0.0345
X_2	Glucose-6-phosphate	1.011
X_3	Fructose-1,6-diphosphate	9.144
X_4	Phosphoenolpyruvate	0.0095
X_5	ATP	1.1278

Enzymes and fixed variables

Symbol	Name	Value
X_6	Glucose uptake (HXT)	19.7 mM min^{-1}
X_7	Hexokinase (GLK)	68.5 mM min^{-1}
X_8	Phosphofructokinase (PFK)	31.7 mM min^{-1}
X_9	Glyceraldehyde-3-phosphate dehydrogenase (GAPD or, as alternative name, TDH)	49.9 mM min^{-1}
X_{10}	Pyruvate kinase (PYK)	3440 mM min^{-1}
X_{11}	Polysaccharide production (glycogen+trehalose)	14.31 mM min^{-1}
X_{12}	Glycerol production	203 mM min^{-1}
X_{13}	ATPase	25.1 mM min^{-1}
X_{14}	NAD^+/NADH ratio	0.042

GMA model

$$\dot{X}_1 = 0.8122 X_2^{-0.2344} X_6 - 2.8632 X_1^{0.7464} X_5^{0.0243} X_7$$
$$\dot{X}_2 = 2.8632 X_1^{0.7464} X_5^{0.0243} X_7 - 0.5232 X_2^{0.7318} X_5^{-0.3941} X_8 - 0.0009 X_2^{8.6107} X_{11}$$
$$\dot{X}_3 = 0.5232 X_2^{0.7318} X_5^{-0.3941} X_8 - 0.0011 X_3^{0.6159} X_5^{0.1308} X_9 X_{14}^{-0.6088} - 0.04725 X_3^{0.05} X_4^{0.533} X_5^{-0.0822} X_{12}$$
$$\dot{X}_4 = 2 \times \left(0.0011 X_3^{0.6159} X_5^{0.1308} X_9 X_{14}^{-0.6088}\right) - 0.0945 X_3^{0.05} X_4^{0.533} X_5^{-0.0822} X_{10}$$
$$\dot{X}_5 = 2 \times \left(0.0011 X_3^{0.6159} X_5^{0.1308} X_9 X_{14}^{-0.6088}\right) + 0.0945 X_3^{0.05} X_4^{0.533} X_5^{-0.0822} X_{10} - 2.8632 X_1^{0.7464} X_5^{0.0243} X_7 - 0.0009 X_2^{8.6107} X_{11} - 0.5232 X_2^{0.7318} X_5^{-0.3941} X_8 - X_5^1 X_{13}$$

<u>GMA model for the heat shock example.</u>

This model is a modification of the previous one. Basically, a reaction has been introduced to account for the flux going into the pentose phosphate pathway. This flux leads to a slight change in the fluxes, which leads to changes in some of the rate-constants. Furthermore, we compute the synthesis of trehalose as a separate reaction, so that we can evaluate this important flux in the adaptive response.Variable names and enzyme values are the same as in the previous case, except for the new variable X_{15} that accounts for the enzyme Glucose 6-phosphate dehydrogenase (G6PDH) in the first step of the pentose phosphate pathway. Further details can be found in the original publications (Curto et al., 1995; Voit & Radivoyevitch, 2000; Vilaprinyo, Alves, & Sorribas, 2006).The equations for the model are:

$$\dot{X}_1 = 0.9023 X_2^{-0.2344} X_6 - 3.1847 X_1^{0.7464} X_5^{0.0253} X_7$$
$$\dot{X}_2 = 3.1847 X_1^{0.7464} X_5^{0.0253} X_7 - 0.5232 X_2^{0.7318} X_5^{-0.3941} X_8 - 0.0009 X_2^{8.6107} X_{11} - 1.76898\, X_2^{0.0526} X_{15}^{0.9646}$$
$$\dot{X}_3 = 0.5232 X_2^{0.7318} X_5^{-0.3941} X_8 - 0.0011 X_3^{0.6159} X_5^{0.1308} X_9 X_{14}^{-0.6088} - 0.0516 X_3^{0.05} X_4^{0.533} X_5^{-0.0822} X_{12}$$
$$\dot{X}_4 = 2 \times \left(0.0011 X_3^{0.6159} X_5^{0.1308} X_9 X_{14}^{-0.6088}\right) - 0.0947 X_3^{0.05} X_4^{0.533} X_5^{-0.0822} X_{10}$$
$$\dot{X}_5 = 2 \times \left(0.0011 X_3^{0.6159} X_5^{0.1308} X_9 X_{14}^{-0.6088}\right) + 0.0947 X_3^{0.05} X_4^{0.533} X_5^{-0.0822} X_{10} - 3.1847 X_1^{0.7464} X_5^{0.0243} X_7 - 0.0009 X_2^{0.7318} X_{11} - 0.5232 X_2^{0.7318} X_5^{-0.3941} X_8 - X_5^1 X_{13}$$

Physiological constraints

The set of constraints in Table 2 of the main manuscript correspond to the threshold values used by (Vilaprinyo et al., 2006). After considering a set of important physiological processes (fluxes of ATP, NADH, Glycerol, Trehalose, metabolite concentrations, cost of changing gene expression, etc.) the numerical values where considered as tentative thresholds that could explain the adaptive response to heat shock in yeast. Details on this election can be found in (Vilaprinyo et al., 2006).

Here, it is important to indicate that the method we present in this paper allows for finding the parameter changes (in terms of changes in the rate-constants) that lead the system to meet a set of numerical constraints. These constraints should result from a sound knowledge of the biological problem and are not produced by our method. However, a systematic analysis of different constraints and the evaluation of the resulting feasibility regions would help in discarding some constraints and in discussing the validity of different sets. Comparison of the feasibility regions obtained by our method and experimental results would finally validate the set of constraints.

The rate of trehalose (see (Voit & Radivoyevitch, 2000) for details of this definition), ATP, NADPH, and Glycerol synthesis are computed as:

Metabolite	Rate of synthesis
Trehalose	$\left(0.00089 X_2^{0.8} X_{11}\right)/11$
NADPH	$1.76898\, \mathrm{X}_2^{0.0526} X_{15}^{0.9646}$
ATP	$2 \times \left(0.0011 X_3^{0.6159} X_5^{0.1308} X_9 X_{14}^{-0.6088}\right) + 0.0947 X_3^{0.05} X_4^{0.533} X_5^{-0.0822} X_{10}$
Glycerol	$0.0516 X_3^{0.05} X_4^{0.533} X_5^{-0.0822} X_{12}$

As in (Vilaprinyo et al., 2006) the cost is defined as the sum of the absolute values of the logarithms of the fold-change for each enzyme, that is $\mathrm{Cost} = \sum_i \left|\ln(k_i)\right|$, where k_i are the fold-changes of each of the considered enzymes.

The index ψ is defined as the product $k_3 k_6$, where k_3 and k_6 are the fold-change in PFK and Polysaccharide production.

References

Curto, R., Sorribas, A., & Cascante, M. (1995) Comparative characterization of the fermentation pathway of saccharomyces cerevisiae using biochemical systems theory and metabolic control analysis: Model definition and nomenclature. *Mathematical biosciences. 130*, 25-50.

Polisetty, P. K., Voit, E. O., & Gatzke, E. P. (2006) Identification of metabolic system parameters using global optimization methods. *Theoretical biology & medical modelling. 3*, 4.

Vilaprinyo, E., Alves, R., & Sorribas, A. (2006) Use of physiological constraints to identify quantitative design principles for gene expression in yeast adaptation to heat shock. *BMC Bioinformatics. 7*, 184.

Voit, E. O., & Radivoyevitch, T. (2000) Biochemical systems analysis of genome-wide expression data. *Bioinformatics. 16*, 1023-37.
